# Metabolism of renin-angiotensin and enkephalin in human follicular fluid: An experimental study

**DOI:** 10.18502/ijrm.v20i10.12269

**Published:** 2022-11-02

**Authors:** Maria Victoria Rodríguez Gallego, Maria Victoria Aparicio Prieto, Andrea Leza García, Juana Hernández Hernández, Jose Antonio Arizaleta Uralde, Luis Casis Sáenz

**Affiliations:** ^1^Clínica Ginecológica Juana, Hernández, Logroño La Rioja Country, Spain.; ^2^Human Reproduction Unit, Cruces University Hospital, Barakaldo (Basque Country), Spain.; ^3^Department of Physiology, Faculty of Medicine and Nursing, University of the Basque Country (UPV/EHU), Bilbao, Spain.

**Keywords:** Follicular fluid, Peptides, Ovarian diseases, Women, Endometriosis, Infertility.

## Abstract

**Background:**

The relationship between the biochemical characteristics of follicular fluid (FF), oocyte quality and embryonic development has not yet been elucidated. We compared samples of FF with a normal metabolic profile against samples with metabolic abnormalities to identify potential predictive biomarkers of reproductive success.

**Objective:**

To analyze peptide activity in the FF of women undergoing in vitro fertilization using 3 samples of FF per individual.

**Materials and Methods:**

FF samples were obtained by ovum pick-up. Pathological samples were defined as samples of FF obtained from women with a gynecological condition or with infertility. A total of 30 women participated in this study. 3 samples of FF were obtained per individual (90 samples), but 8 samples were excluded because they were hemolyzed. The samples (n = 82 FF) included controls (n = 36, donors without fertility problems), women with endometriosis (n = 15), unexplained infertility (n = 19), and aged > 39 (n = 12). We assessed local encephalinergics: aminopeptidase-N (puromycin sensitive aminopeptidase and neutral endopeptidase; and components of the angiotensin system of the reproductive tract: prolyl-endopeptidase, APN, aspartate-aminopeptidase, and basic-aminopeptidase.

**Results:**

No differences were observed in peptide metabolism based on the presence or absence of oocytes in the FF. Women with endometriosis and aged > 39 yr showed alterations in puromycin sensitive aminopeptidase (p = 0.01), aminopeptidase-B (p = 0.01), aspartate-aminopeptidase (p < 0.001) and neutral endopeptidase (p < 0.001).

**Conclusion:**

This study reveals alterations in the metabolism of enkephalin and angiotensin in pathological FF, which points to these components as potential diagnostic biomarkers.

## 1. Introduction

The follicular fluid (FF) is produced from blood plasma components and secretions from the granulosa and thecal cells (1). FF provides the microenvironment for oocyte maturation (2). Indeed, the follicular microenvironment will determine whether a follicle is selected or not for ovulation (3). Adequate folliculogenesis requires metabolic stability, and alterations in follicular development may induce a premature loss of reproductive capacity. Therefore, FF biochemistry may determine oocyte quality and the potential to achieve successful fertilization and embryonic development (4-6). To test this hypothesis, we developed a preliminary study to examine whether the biochemical composition of FF remains stable or presents alterations that could influence oocyte quality. In fact, in the last 50 yr, several studies have assessed the potential relationship between the composition of FF and reproductive outcomes (7-9). In recent years, several peptidergic systems have been associated with the regulatory processes of male and female fertility (10, 11). These systems include local endogenous opioidergic systems (12, 13) (gamma-endorphin, beta-endorphin, met-enkephalin, immunoreactive beta-lipotropin), and components of the renin-angiotensin system (14, 15) (angiotensin 1-8, angiotensin 1-7 or angiotensin II). Their distinct cellular localization pattern in human ovarian tissue during folliculogenesis and in luteal tissue suggests a role in the growth and differentiation of luteal, granulosa and theca cells (5).

FF is easily accessible during ovum pick-up and can be aspirated along with the oocyte. To explore the correlation between FF biochemistry and oocyte quality, each follicle must be aspirated separately (6). The reason is that metabolic differences may be observed in FF samples of the same individual obtained from separate aspirations.

In this study, we analyzed peptide activity in the FF of women undergoing in vitro fertilization. To such purpose, 3 samples of FF were analyzed per individual. The study variables included the presence/absence of oocyte in the FF, the age of the women, and conditions related/unrelated to ovarian diseases, i.e., endometriosis and sterility of unknown etiology, known as unexplained infertility (16, 17). To assess peptidergic metabolic stability, we assessed local encephalinergics: aminopeptidase-N (neutral aminopeptidase, APN), puromycin sensitive aminopeptidase (PSA) and neutral endopeptidase (NEP); and components of the renin-angiotensin system of the reproductive tract: prolyl endopeptidase (PEP), which converts angiotensin I (AI) and AII to A1-7; APN, which converts AIII to AIV; aspartate-aminopeptidase (Asp-AP), which transforms AII to AIII; and basic aminopeptidase (aminopeptidase B or APB), with converts AIII to AIV. The aim was twofold: i) to assess whether the biochemical composition of FF remains stable or otherwise presents alterations, and ii) to better understand the role of these enzymes and their substrates in follicular physiology (or pathophysiology, in the case of infertility). Finally, we aimed to explore potential biomarkers that are useful in predicting and assessing female infertility and help improve reproductive outcomes.

## 2. Material and Methods

### Study population

This was an experimental study. FF samples were obtained from women aged 19-42 yr undergoing a fertility treatment in the In Vitro Fertilization Unit of the Gynecological Clinic of Dr. Juana Hernández (Logroño, Spain). Different inclusion criteria were applied to each group. The control group included oocyte donors between 19 and 31 yr of age without known diseases. The endometriosis group (END group) included women aged 31-39 with an established diagnosis of endometriosis. The group of unexplained infertility (UI group) was composed of women aged 33-38 yr with infertility of unknown etiology diagnosed after an infertility work-up. The > 39-yr group consisted of women aged 39 yr or more without known diseases. The exclusion criteria included age < 18 or > 43 yr, or declining to participate in the study.

A total of 30 women participated in this study. 3 samples of FF were obtained per individual (90 samples). 8 samples were excluded from the analysis because they were hemolyzed, so the total number of samples in our study was 82. The samples of FF were categorized into 4 groups: controls (n = 36, donors without fertility problems); END (n = 15); > 39 (n = 12); and UI (n = 19). The sample volume obtained (minimum 3 ml) was always much higher than the necessary amount to make biochemical determinations.

### FF collection

FF samples were obtained by ovum pick-up. During the procedure, 3 random follicles were sequentially aspirated and marked. Then, aspirates were sent to the laboratory of embryology to assess the presence of oocytes in the FF samples. Immediately after oocyte retrieval, the FF was stored at -80°C for later analysis of enzymatic activity. Cautionary measures were adopted to avoid contamination of the fluid samples with blood. The FF samples were thawed to eliminate impurities and centrifuged at 3000 g at 4°C for 5 min. Later, the supernatant was removed, and specific enzymatic activity was assessed. The study variables included the presence or absence of oocytes in the FF, the age of the women, and diseases related/unrelated to ovarian disorders (endometriosis and sterility of unknown etiology).

### FF metabolic activities

We assessed the local metabolism of enkephalins, including APN, PSA, and NEP. The local angiotensin renin system was also analyzed, including PEP, which converts AI and AII to A1-7; APN, which converts AIII to AIV; Asp-AP, which hydrolysis AII to AIII; and APB, which converts AIII to AIV. By this method, we analyzed the complete metabolic degradation of peptidases in the 2 systems.

Aminopeptidase activity was measured by fluorometry according to previously described methods (18). The assay is based on the fluorescence of products generated from the hydrolysis of a specific substrate by each enzyme present in the sample. Several aminoacyl-2-naphthylamide derivatives (Sigma Aldrich, St. Louis, MO, USA) were used as enzymatic substrates. Substrate solutions were prepared in a 50 mM phosphate buffer (pH 7.4) containing 0.25 mg/ml of bovine serum albumin for APN (0.5 mM), APB (0.5 mM; pH 6.5), APA (0.125 mM) and PEP (0.125 mM), and in a 50 mM Tris-HCl buffer (pH 7.4) containing 0.25 mg/ml of bovine serum albumin for NEP.

Reactions were triggered by adding 10 μl of the FF sample to 1 ml of the incubation mixture with fluorogenic-derived substrates (0.125 mM aminoacyl-β-naphthylamide). Alanine aminopeptidase activities (APN and PSA) were measured using Ala-β-naphthylamide as substrate.

Incubations with the specific PSA inhibitor puromycin (40 μM) (Sigma Aldrich, St. Louis, MO, USA) were performed in parallel to discriminate APN and PSA activity from total alanine aminopeptidase activity. APB (basic aminopeptidase) and Asp-aminopeptidase activity were quantified based on Arg-β-naphthylamide and Asp-β-naphthylamide levels. PEP activity was assayed using Z-Gly-Pro-β-naphthylamide (Bachem, CA, USA) (18, 19).

N-Dansyl-D-Ala-Gly-p-Nitro-Phe-Gly (DAGNPG) (Sigma Aldrich, St Louis, MO, USA) was used as a fluorogenic substrate to measure NEP activity (19, 20). The substrate solutions for PSA, APN, and PEP (pH 7.4) and for APB (pH 6.5) were prepared in a 50 mM phosphate buffer solution. Asp-AP activity was assayed in Tris-HCl buffer (50 mM; pH 5.9). After incubation (30 min, 37°C), the enzymatic reaction was stopped by adding 1 ml of 0.1 M sodium acetate buffer (pH 4.2). The released β-naphthylamine was determined by measuring the fluorescence intensity in the reaction mixture at 412 nm (with an excitation wavelength of 345 nm) using a spectrofluorometer RF540, Shimadzu, Japan. To determine the released DAGNPG in the NEP activity assay, fluorescence was measured at 410 nm and 342 nm excitation. Fluorescence was converted to pmol of product using a standard curve made with increasing concentrations of β-naphthylamine or decreasing concentrations of DAGNPG (Sigma Aldrich, St. Louis, MO, USA).

All assays were performed in triplicate. Results were expressed as units of peptidase activity per liter of sample: UP/L. A UP is the enzyme that hydrolyzes 1 pmol of fluorogenic substrate per min.

### Ethical considerations

This study was carried out in accordance with the international standards on clinical trials. In the preliminary interview, women were made aware that participation was entirely voluntary, were informed about the anonymity and confidentiality of the data, and read and signed an informed consent form. The study protocol was reviewed and approved by the Ethics Committee of the Quirón Clinic hospitals, Spain (Code: GHQZ 01/06/08).

### Statistical analysis

Differences between the quantitative variables were assessed using student's *t* test for independent groups. When a statistically significant difference was obtained through the Shapiro-Wilk test, the Mann-Whitney U test was performed. Repeated measures ANOVA with Sidak adjustment for multiple comparisons was used to compare the means across 1 or more variables; the purpose was to assess differences in peptide activity across the 3 FF samples of the same individual. All statistical tests were bilateral, considering 95% confidence intervals. All statistical analyses were performed using the Statistical Package for the Social Sciences (SPSS), version 21.0 (SPSS Inc., Chicago, IL, USA) and the R Commander 3.3.3 software package. Significant difference is considered from p < 0.05.

## 3. Results

**Figure 1:**
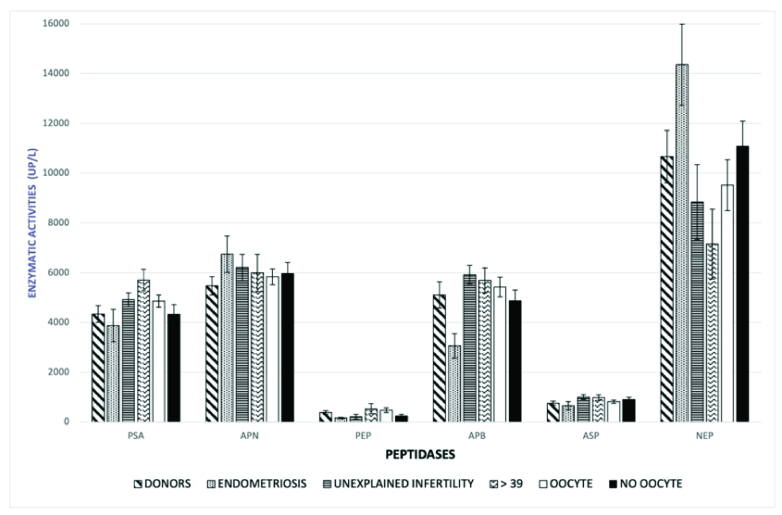
Enzymatic activity of 6 peptidases in FF by study group, including the presence or absence of an oocyte. The statistical mean for each enzyme is presented. Enzymatic activity is expressed as UP/L (pmol of hydrolyzed fluorogenic substrate/min/liter of the sample). PSA: Puromycin sensitive aminopeptidase, APN: Aminopeptidase-N, PEP: Prolyl-endopeptidase, APB: Basic-aminopeptidase, ASP: Aspartate-aminopeptidase, NEP: Neutral endopeptidase.

We assessed the enzymatic activity of 6 peptidases in the FF of 4 groups of women: donors (control group), women with endometriosis (END group), women with unexplained infertility (UI group), and women aged > 39 yr ( > 39 group). The enzymes analyzed included APN, PSA, PEP, Asp-AP, APB, and NEP (Figure 1). The peptidases with the highest activity were NEP and APN, whereas PEP showed the lowest activity. Figure 1 also shows the enzymatic activity of the 6 peptidases according to the presence or absence of an oocyte. As observed, the 3 samples of FF of each individual showed similar peptidase activity, regardless of whether they contained an oocyte, without statistically significant differences. The peptidase with the highest enzymatic activity was NEP, followed by APN, APB, and PSA, whereas PEP showed the lowest activity. Within-subject variations were not observed, and no visible correlations were found. In summary, there were no statistically significant differences in the mean enzymatic activity in the FF samples according to the presence or absence of an oocyte.

**Figure 2:**
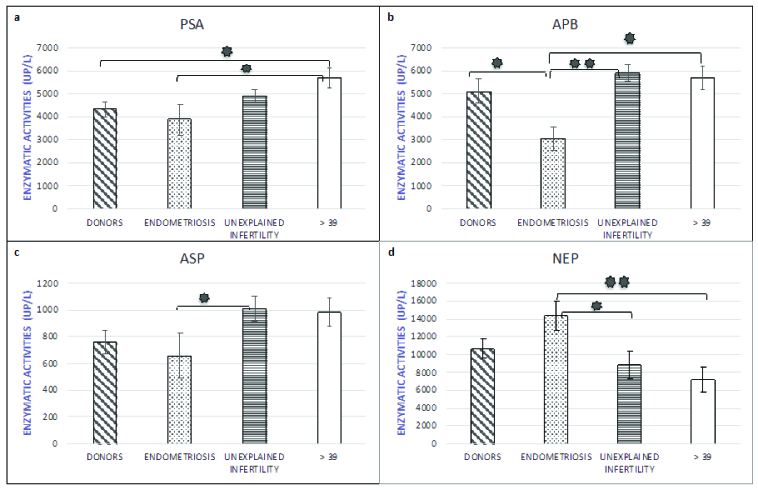
Results of the peptidases in which statistically significant differences were observed across groups: 2a: Puromycin sensitive aminopeptidase (PSA), 2b: Basic-aminopeptidase (APB), 2c: Asp-aminopeptidase (ASP), 2d: Neutral endopeptidase (NEP). *P < 0.05, **P < 0.01.

Table I shows the results obtained for the comparisons between the groups in the statistical analysis. Figure 2 summarizes the results for the peptidases that showed statistically significant differences across the groups. PSA activity was significantly increased in the END and age > 39 groups (Figure 2a). PSA activity in the > 39 group was 1.5 times higher than in the END group, which was a statistically significant difference (p = 0.018). Consistently, PSA activity was 1.3 times lower in the control group (donors), as compared to in the > 39 group, which was a statistically significant difference (p = 0.033).

Figure 2b shows the APB activity in the different study groups. APB activity was significantly reduced in END with statistically significant differences with all groups. As compared to the UI and age > 39 groups, APB activity was 2 times lower in the END group (END vs. UI, p = 0.007; END vs. > 39 group, p = 0.013). The differences with respect to the donors were slightly lower, with APB activity being 1.7 times higher in the donors than in the END group (p = 0.031). All differences were statistically significant.

Figure 2c shows Asp-AP activity by study group. The lowest Asp-AP activity was observed in the END group, whereas the group with the highest Asp-AP activity was UI, followed by the > 39 group. Asp-AP activity was 1.5 times higher in the UI group than in the END group, and this difference was statistically significant (p = 0.049).

The group with the most elevated NEP activity was the END group, whereas the lowest NEP activity was observed in the > 39 group (Figure 2d). NEP activity was 2 times lower in the > 39 group than in the END group, and this difference was statistically significant (p = 0.003). Similarly, NEP activity was 1.6 times lower in the UI group than in the END group, which was a statistically significant difference (p = 0.019).

**Table 1:** Mean comparisons between the groups


**Dependent variables**			**Difference in means (I-J)**	**Standard error**	**P-value**
**PSA**					
	**Control**				
	**END**	459.92859	600.14713	0.45
	**UI**	-573.85845	546.74672	0.30
	** > 39 yr**	-1364.29734 ${}^{*}$	624.23130	0.03
	**Endometriosis**				
	**Control**	-459.92859	600.14713	0.45
	**UI**	-1033.78703	685.23130	0.14
	** > 39 yr**	-1824.22592 ${}^{*}$	748.51498	0.02
	**Unexplained infertility**				
	**Control**	573.85845	546.74672	0.30
	**END**	1033.78703	685.23130	0.14
	** > 39 yr**	-790.43889	706.42061	0.27
	** > 39 yr**				
	**Control**	1364.29734 ${}^{*}$	624.23130	0.03
	**END**	1824.22592 ${}^{*}$	748.51498	0.02
	**UI**	790.43889	706.42061	0.27
**APB**					
	**Control**				
	**END**	2050.82861 ${}^{*}$	932.08567	0.03
	**UI**	-816.33043	721.99046	0.26
	** > 39 yr**	-584.86755	737.86028	0.43
	**Endometriosis**				
	**Control**	-2050.82861 ${}^{*}$	932.08567	0.03
	**UI**	-2867.15904 ${}^{*}$	1021.04870	0.01
	** > 39 yr**	-2635.69616 ${}^{*}$	1032.33135	0.01
	**Unexplained infertility**				
	**Control**	816.33043	721.99046	0.26
	**END**	2867.15904 ${}^{*}$	1021.04870	0.01
	** > 39 yr**	231.46289	847.46358	0.79
	** > 39 yr**				
	**Control**	584.86755	737.86028	0.43
	**END**	2635.69616 ${}^{*}$	1032.33135	0.01
	**UI**	-231.46289	847.46358	0.79
**ASP**					
	**Control**				
	**END**	100.24150	161.89368	0.54
	**UI**	-243.97408	130.62017	0.07
	** > 39 yr**	-222.55680	140.61340	0.12
	**Endometriosis**				
	**Control**	-100.24150	161.89368	0.54
	**UI**	-344.21558 ${}^{*}$	171.68776	0.05
	** > 39 yr**	-322.79830	179.40787	0.08
	**Unexplained infertility**				
	**Control**	243.97408	130.62017	0.07
	**END**	344.21558 ${}^{*}$	171.68776	0.05
	** > 39 yr**	21.41728	151.78685	0.89
	** > 39 yr**				
	**Control**	222.55680	140.61340	0.12
	**END**	322.79830	179,40787	0.08
	**UI**	-21.41728	151.78685	0.89
**NEP**					
	**Control**				
	**END**	-3691.15624	1961.06440	0.06
	**UI**	1828.94419	1845.39165	0.33
	** > 39 yr**	3516.23649	1899.28302	0.07
	**Endometriosis**				
	**Control**	3691.15624	1961.06440	0.06
	**UI**	5520.10042 ${}^{*}$	2298.61672	0.02
	** > 39 yr**	7207.39273 ${}^{*}$	2342.10258	< 0.001
	**Unexplained infertility**				
	**Control**	-1828.94419	1845.39165	0.33
	**END**	-5520.10042 ${}^{*}$	2298.61672	0.02
	** > 39 yr**	1687.29230	2246.13919	0.46
	** > 39 yr**				
	**Control**	-3516.23649	1899.28302	0.07
	**END**	-7207.39273 ${}^{*}$	2342.10258	< 0.001
	**UI**	-1687.29230	2246.13919	0.46
PSA: Puromycin sensitive aminopeptidase, APB: Aminopeptidase B, ASP: Aspartate, NEP: Neutral endopeptidase, END: Endometriosis; UI: Unexplained infertility, yr: Year, * p < 0.05					

## 4. Discussion

The present study sought to describe the activity of enkephalin-degrading enzymes (PSA, APN, and NEP) and renin-angiotensin system enzymes (PEP: AI and AII to A1-7; APN: AIII to AIV; Asp-AP: AII to AIII; APB: AIII to AIV) in human FF. The purpose was to better understand the role of these enzymes and their substrates in follicular physiology (or pathophysiology in the case of infertility). Secondly, we aimed to explore potential biomarkers that help predict or assess female infertility and can contribute to improved reproductive outcomes. FF components emerge as potential predictors of oocyte quality. We described and compared enzymatic activity in FF based on the presence or absence of an oocyte. We also analyzed these metabolisms in relation to the presence/absence of conditions such as endometriosis and unexplained infertility.

The reason why some FF samples contain an oocyte while others do not is unclear. Some authors have posited that the oocyte could remain in residual FF when FF is partially aspirated (21). Another reason could be that oocytes with complex aneuploidies remain strongly adhered to the follicular wall, thus resisting aspiration (22). Borderline empty follicle syndrome could explain why, in some cycles, the number of oocytes retrieved does not fit the number of follicles counted by ultrasound. Empty follicle syndrome has been described, and several hypotheses have been posited, including error in drug administration, advanced age, long-standing infertility, low estrogen levels, and endometriosis, to name a few (23-25). Some researchers have suggested an intrinsic ovarian alteration or a genetic etiology. In any case, assessing whether the presence or absence of an oocyte in FF is associated with biochemical alterations in the FF would shed some light. Since 3 FF samples were obtained from each woman in this study, a twofold method was employed for comparison of the results.

Firstly, we explored the potential correlation between the presence/absence of an oocyte and peptidase activity in the 3 FF samples of each woman. Individual data do not show appreciable differences between them thus, significant differences were not observed in the mean enzymatic activity between oocyte-containing FF and empty FF. There were no clear within-subject differences in peptide metabolism across the 3 individual FF samples, regardless of them containing a gamete or not. This means that antral fluid containing oocytes remained stable and gamete-containing FF was not affected. Therefore, none of the enzymes assayed emerged as a potential biomarker.

Female fertility peaks at 20-24 yr and progressively decreases over time (7, 26, 27). The pregnancy rate per in vitro fertilization cycle reaches near 40% at 34 yr and drops dramatically in the following 5 yr, a tendency that remains stable throughout reproductive life. The pregnancy rate at age 45 is virtually 0, since it is near menopausal age. We compared enzyme levels in 2 age groups: women older and younger than 39 yr. Differences in activity levels were also assessed across the 3 samples obtained from each woman. Like in the case of the presence or absence of a gamete, individual data did not show any correlation. There were no clear differences in the enzymatic activity across the 3 FF samples of each woman. The behavior of several peptidases in the FF of fertile women categorized into 5 age ranges has been previously described (11). The study did not reveal significant age-based differences in the activity of APN, dipeptidyl peptidase IV, glu-aminopeptidase, PEP or pGlutamyl-aminopeptidase I. In contrast, statistically significant differences were found in PSA, APB, Cys-aminopeptidase, and Asp-AP from 40 yr of age. In the same line, the mean levels of enzymatic activity in the FF samples of women < 39 vs. > 39 (2 groups) revealed statistically significant differences in PSA (the highest difference) and APB between the groups. To the best of our knowledge, this is the first study to uncover a significant decrease of NEP in women > 40. This finding confirms PSA as a potential biomarker of follicular quality and, consequently, of oocyte quality. This is consistent with several other studies which have documented that the activity of important proteases involved in peptide metabolism increases as the age of the tissue increases (27).

To analyze the potential effect of diverse conditions on FF composition, we considered those most strongly related to ovarian alterations. In this case, we assessed metabolic activity in women with endometriosis as compared to fertile donors or women with unexplained infertility, which affects couples without any apparent fertility problems. Endometriosis is defined as endometrial tissue out of the uterine cavity. Endometriosis has been posited to be associated with poorer oocyte quality owing to an adverse follicular environment, thereby resulting in lower fertilization, embryonic development, and implantation rates (16). In our study, comparison of enzymatic levels per individual did not show any correlation. We found, however, statistically significant differences in the total mean values for APB between the control group and the END group. There were statistically significant differences between the UI and the END groups in terms of APB, Asp-AP, and NEP activity. We found statistically significant differences between the controls and the UI group as well. These results confirm the null hypothesis: there are no differences in the metabolism of follicular peptides between donors (fertile women without conditions) and women with unexplained infertility, since the gamete microenvironment remains stable. This contrasts with the behavior of FF in women with endometriosis, who show alterations in virtually all the peptides studied (as the local opioidergic system is especially affected).

## 5. Conclusion 

The results obtained in this study suggest that an adverse follicular environment shows alterations in the metabolism of enkephalins and angiotensins. Therefore, some of these peptides can be used as diagnostic biomarkers. However, due to the limitations of the study (i.e., the limited number of samples), further studies are needed to verify the role of these peptides in FF stability and to validate the value of FF components as predictive biomarkers of reproductive success.

##  Conflict of Interest 

The authors declare that there is no conflict of interest.
